# Forward and reverse mapping for milling process using artificial neural networks

**DOI:** 10.1016/j.dib.2017.10.069

**Published:** 2017-11-04

**Authors:** Rashmi L. Malghan, Karthik Rao M C, Arun Kumar Shettigar, Shrikantha S. Rao, R.J. D’Souza

**Affiliations:** a Department of Mechanical Engineering, National Institute of Technology, Surathkal, Karnataka, India; b Department of Mechatronics Engineering, Manipal Institute of Technology, Manipal, India; c Department of Mathematical and Computational Science, National Institute of Technology, Surathkal, Karnataka, India

**Keywords:** ANN, Forward mapping, Reverse mapping, Milling process

## Abstract

The data set presented is related to the milling process of AA6061-4.5%Cu-5%SiCp composite. The data primarily concentrates on predicting values of some machining responses, such as cutting force, surface finish and power utilization utilizing using forward back propagation neural network based approach, i.e. ANN based on three process parameters, such as spindle speed, feed rate and depth of cut.The comparing reverse model is likewise created to prescribe the ideal settings of processing parameters for accomplishing the desired responses as indicated by the necessities of the end clients. These modelling approaches are very proficient to foresee the benefits of machining responses and also process parameter settings in light of the experimental technique.

**Specifications Table**

**Table:** 

Subject area	Mechanical engineering, Prediction Techniques
More specific subject area	Prediction Technique/ Milling operation
Type of data	Table, graph, figures
How data was acquired	Data was acquired by experimental techniques
Data format	Raw, and analyzed
Experimental factors	Milling –Metal cutting process
Experimental features	ANN forward and reverse mapping implementation in Milling operation to predict the benefits of machining responses and also process parameter settings in light of the experimental technique.
Data source location	Surathkal, Manipal, India;
Data accessibility	Data is available with this article

**Value of the data**

• This dataset shows the evidence of prediction through forward and reverse mapping through ANN.
• The reverse model dataset will help in the ideal (optimal) settings of processing parameters for accomplishing the desired responses as indicated by the necessities of the end clients.
• This dataset can be used to compare the theoretical and predicted results with other predictive techniques.
• The input and output data, can be further exported by different mathematical models and evolutionary techniques.
• The dataset can be used to predict the other responses in the milling operation.

## 1 Data

Data presented in the article is pertaining to AA6061-4.5%Cu-5%SiCp composite. Using this data an endeavor is made to build up a savvy framework to set up the info yield relationship of a processing procedure while using forward and reverse mappings of simulated neural systems (ANNs). In forward mapping, cutting force, surface finish, and power consumption is anticipated from a known arrangement of three machining parameters, namely, spindle speed, feed rate and depth of cut. An endeavor is likewise made to build up the comparing reverse model to foresee the suggested procedure parameter settings for accomplishing the coveted reactions to meet the end client's prerequisites. Toward this path, a back propagation neural network (BPNN)- based approach is connected to build up the related ANN models. In training, a batch mode is utilized in both the directed learning systems which require an extensive arrangement of preparing information.This requirement for having a large set of training data is fulfilled by artificially generating the necessary data with the help of simulation based on the real time experimental observations of the earlier researchers [1]. The performance of BPNN is also validated against the past experimental data to show its effectiveness and suitability in advanced machining applications in selecting the settings of the most influential process parameters to achieve the desired responses.

## 2 Experimental design, materials and methods

### 2.1 Test equipment

The experiments on a vertical CNC milling machine and investigated the effects of three process parameters, i.e. spindle speed (rpm), feed rate (mm/min) and depth of cut (mm) on three process responses, like cutting force (N), surface roughness (µm) and power consumption (kW) [1]. Each of the process parameters was set at three different levels, i.e. spindle speed at 1000 rpm, 2000 rpm and 3000 rpm; feed rate at 300 mm/min, 400 mm/min and 500 mm/min; depth of cut at 1 mm, 2 mm and 3 mm. A aluminium based composite material, i.e. AA6061-4.5%Cu-5%SiCp was considered for the experimental work. The detailed experimental plan along with the observed responses is represented in Table 1.

**Table 1:** Experimental data V/S ANN predicted results and prediction error for forwarding mapping.

**Experimental Data**							**ANN Prediction**			**Prediction Error (%)**		
**Trial. No**	**Spindle Speed (rpm)**	**Feed Rate (mm/min)**	**Depth of cut (mm)**	**Cutting Force, F(N)**	**Surface Roughness, Ra (µm)**	**Power Consumption (kW)**	**ANN F**	**ANN Ra**	**ANN Power**	**F Error**	**Ra Error**	**Power Error**
1	1000	300	1	71.28	3.24	0.06	69.88	3.19	0.06	1.96	1.50	-0.90
2	1000	300	2	78.03	2.99	0.07	80.01	3.04	0.07	-2.53	-1.58	-0.58
3	1000	300	3	87.64	2.35	0.08	89.02	2.33	0.08	-1.58	0.87	-0.42
4	1000	400	1	90.37	3.81	0.11	91.13	3.87	0.11	-0.85	-1.70	-3.81
5	1000	400	2	109.45	3.39	0.13	107.30	3.36	0.13	1.96	1.02	0.34
6	1000	400	3	115.33	2.70	0.14	116.88	2.75	0.14	-1.34	-1.94	-1.18
7	1000	500	1	120.39	4.35	0.18	117.50	4.28	0.19	2.40	1.64	-2.43
8	1000	500	2	134.79	3.99	0.20	131.86	3.96	0.20	2.18	0.66	1.96
9	1000	500	3	139.53	3.06	0.21	139.57	3.07	0.21	-0.03	-0.18	0.39
10	2000	300	1	144.91	2.00	0.15	147.25	2.01	0.15	-1.62	-0.63	-1.18
11	2000	300	2	155.12	2.07	0.15	158.35	2.06	0.16	-2.09	0.39	-2.77
12	2000	300	3	176.66	1.77	0.17	170.70	1.73	0.17	3.38	2.44	0.88
13	2000	400	1	179.40	2.44	0.23	174.68	2.43	0.23	2.63	0.32	1.93
14	2000	400	2	182.50	2.29	0.24	177.74	2.24	0.24	2.61	2.13	0.42
15	2000	400	3	185.55	1.74	0.24	185.05	1.71	0.25	0.27	1.45	-0.54
16	2000	500	1	187.34	2.82	0.31	187.86	2.77	0.31	-0.27	1.86	0.29
17	2000	500	2	190.09	2.58	0.32	194.86	2.63	0.32	-2.51	-1.75	-0.50
18	2000	500	3	194.46	1.94	0.33	198.14	1.98	0.33	-1.90	-1.86	0.39
19	3000	300	1	232.49	1.20	0.24	235.83	1.21	0.24	-1.44	-1.03	0.49
20	3000	300	2	241.82	1.40	0.25	240.30	1.37	0.25	0.63	2.04	0.76
21	3000	300	3	245.12	1.30	0.25	244.05	1.33	0.25	0.44	-2.47	0.23
22	3000	400	1	247.98	1.21	0.33	245.33	1.21	0.33	1.07	-0.35	-0.60
23	3000	400	2	249.63	1.34	0.34	248.86	1.32	0.34	0.31	1.32	-0.67
24	3000	400	3	250.16	1.14	0.35	251.74	1.13	0.35	-0.63	0.94	-0.45
25	3000	500	1	254.03	1.45	0.44	254.93	1.49	0.44	-0.35	-2.44	0.06
26	3000	500	2	257.86	1.35	0.44	257.59	1.38	0.44	0.11	-2.23	0.27
27	3000	500	3	259.00	1.00	0.45	259.68	1.02	0.45	-0.26	-1.64	0.17

### 2.2 Method: parametric study of forward mapping - back propagation neural network (BPNN) specification

**Fig. 1:**
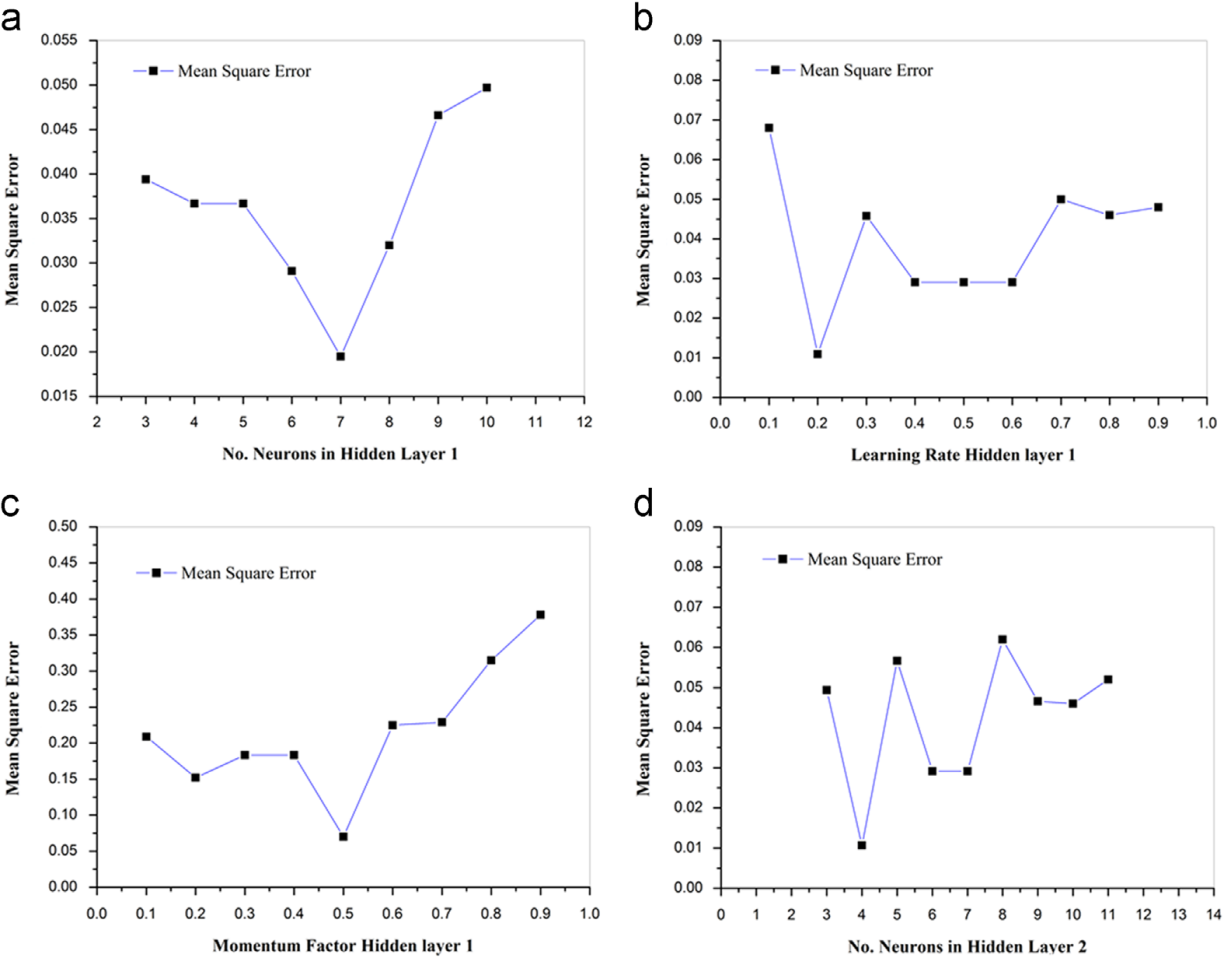
Depicts Effect of the parametric study to recognize optimal neural network parameters in case of forward mapping: a) Error v/s number of neurons in hidden layer 1. b) Error v/s learning rate in hidden layer 1 and 2 c) Error v/s momentum factor in hidden layer 1 and 2 d) Error v/s number of neurons in hidden layer 2.

**Fig. 2:**
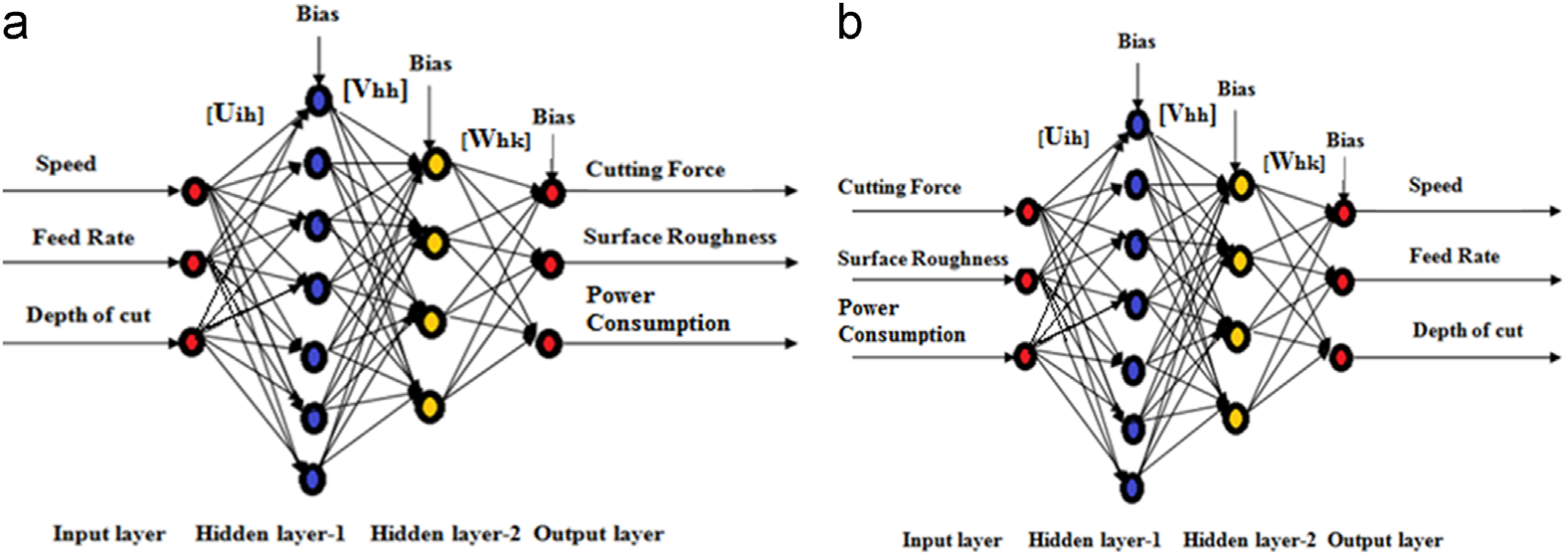
ANN – neural network structure used in case of forward mapping (a) ANN forward mapping (b) ANN reverse mapping.

The Choice of the ideal ANN design to be utilized to forecast is typically chosen by trial strategy, picking the one which contributes the most minimal estimation of mean square mistake (MSE) [3]. The variety of MSE esteems with varying node numbers in the hidden layer1, learning rate,momentum factor and shrouded layer 2 is shown in Fig. 1. Among a few ANN architectures attempted, it is discovered that the 3–7-4-3 architecture, as appeared in Fig. 2, gives the base MSE value. The regulated learning procedure of an ANN for the most part requires a huge set of training data. In actual practice, this necessity of large data is fulfilled by generating artificial datasets through using response equations inferred through test completed before by the same authors [1]. For this situation, in view of the experimentation information of Table 1, 1500 new datasets are created for the preparation reason. This preparation information is then linear normalized to accomplish better training and prediction outcomes. The points of interest of the created ANN show for predicting the responses for a given set of milling process parameters in forward mapping are given as underneath.

• Number of neurons in hidden layer 1:7
• Number of neurons in hidden layer 2:4
• Learning rate factor value: 0.2
• Momentum factor value: 0.5
• Transfer Function used: Sigmoid
• Number of data used for training: 20
• Number of data used for testing: 15.

After the training stage utilizing the new 1500 datasets, the created ANN is utilized for forward and reverse mapping purposes [2].The Forward mapping manages anticipating the reaction of the processing procedure for known arrangement of information conditions. It along these lines satisfies the end client's prerequisites of accomplishing the coveted reactions for differing benefits of processing process parameters. In forward mapping, the end client may likewise acquire the conditional reaction esteems for an obscure arrangement of processing process parameters. Table 1 exhibit the experimentally observed and ANN predicted milling response values along with the estimated prediction error for the considered milling process.

**Fig. 3:**
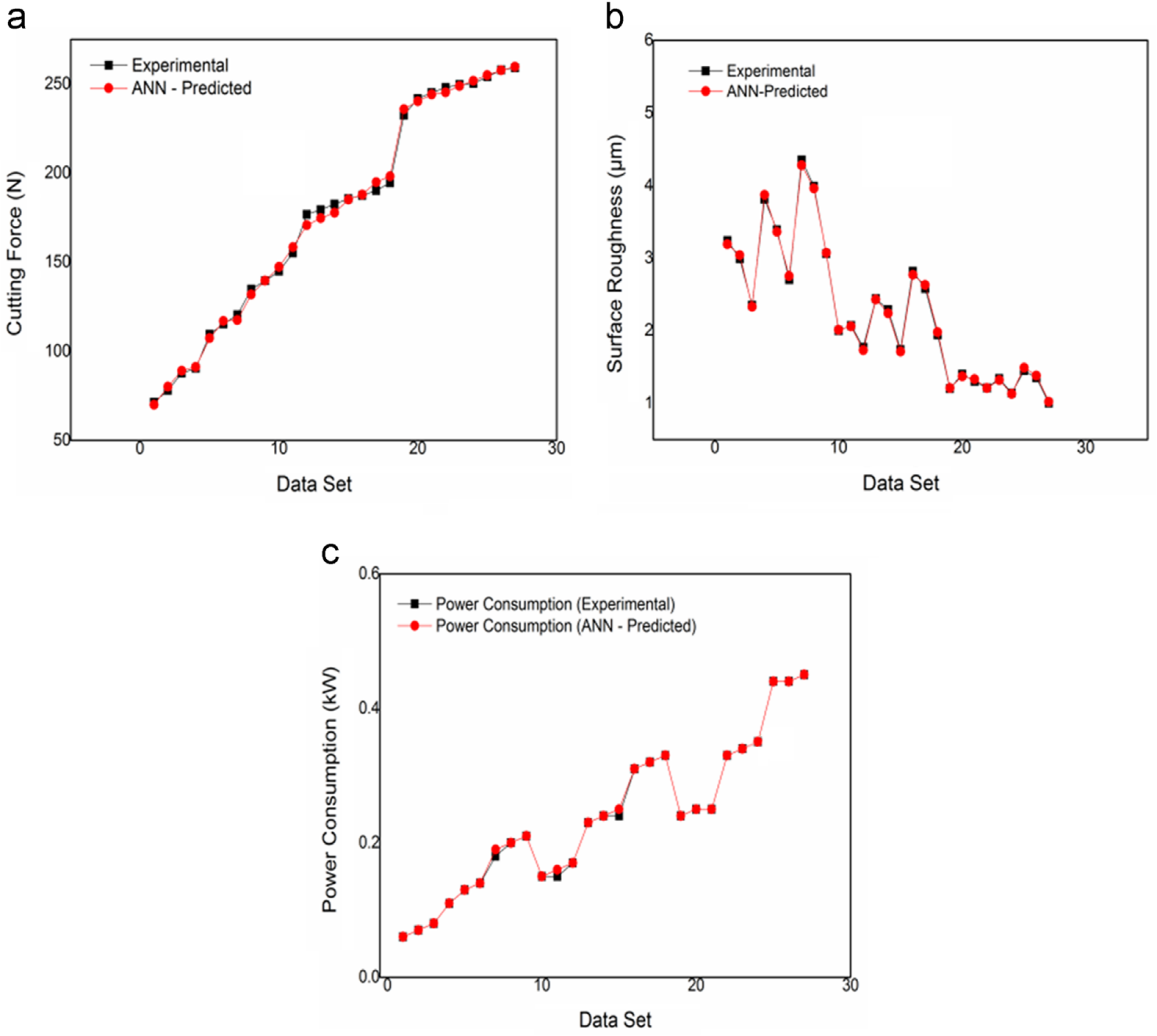
(a-c). Forward mapping ANN predicted V/S experimental (a) Cutting force (b) Surface roughness (c) Power consumption.

**Fig. 4:**
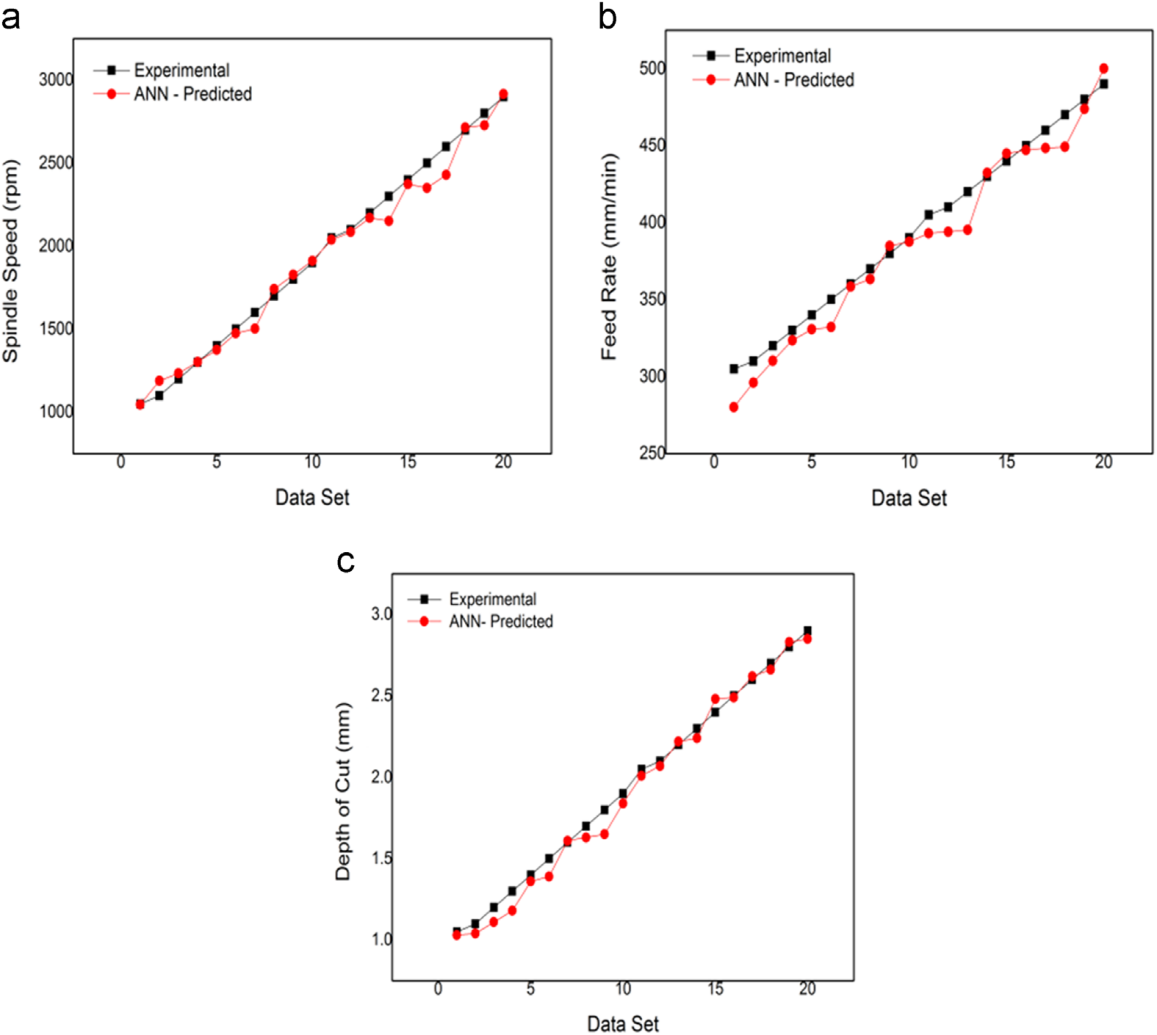
(a-c). Reverse mapping ANN predicted V/S experimental (a) Spindle speed (b) Feed rate (c) Depth of cut.

Fig. 3(a-c) compares the experimental and ANN predicted values of cutting force, surface roughness and power consumption respectively in the considered milling process and it is interesting to observe that for all the three responses, the ANN predicted responses closely match with those obtained experimentally. It is also observed that the relative prediction errors for cutting force lies between, surface roughness and power consumption are only 3.38% to -2.53, 2.44%, to -2.41% and 1.96% to -3.81% respectively, which confirm the developed ANN model to almost accurately predict the output responses for a given set of milling process parameters. An ANN model is also developed for reverse mapping of the considered milling process based on the same (3–7-4-3) ANN architecture. From the Fig. 4(a-c) it can be observed that the ANN predicted value are in good agreement with the experimental values. This model for reverse mapping is also trained using the test cases and is subsequently used for prediction of the tentative settings of the milling process parameters based on a set of desired response characteristics. It can also be treated as an advisory system in the absence of human experts, can predict the settings of various process parameters in milling set up in order to achieve the desired responses according to the requirements of the end users. Table 2 provides a set of 20 test cases, data used for training of the developed ANN for reverse mapping. From Table 2, it is observed that the relative prediction errors in the three milling process parameters, i.e spindle speed, feed rate and depth of cut lies within |10%|.

**Table 2:** Summary of the test cases results for the response: reverse mapping.

**Experimental Data**				**ANN Predicted**			**Error (%)**		
**Test Cases**	**Spindle Speed (rpm)**	**Feed Rate (mm/min)**	**Depth of Cut (mm)**	**Spindle Speed**	**Feed Rate**	**Depth of Cut**	**Spindle Speed**	**Feed Rate**	**Depth of Cut**
1	1050	305	1.05	1046.38	280.22	1.03	0.345	8.125	1.905
2	1100	310	1.1	1189.23	296.13	1.04	-8.112	4.474	5.455
3	1200	320	1.2	1234.17	310.22	1.11	-2.848	3.056	7.500
4	1300	330	1.3	1302.04	323.48	1.18	-0.157	1.976	9.231
5	1400	340	1.4	1375.12	330.61	1.36	1.777	2.762	2.857
6	1500	350	1.5	1474.33	332.07	1.39	1.711	5.123	7.333
7	1600	360	1.6	1502.45	358.35	1.61	6.097	0.458	-0.625
8	1700	370	1.7	1740.52	363.24	1.63	-2.384	1.827	4.118
9	1800	380	1.8	1827.69	384.86	1.65	-1.538	-1.279	8.333
10	1900	390	1.9	1912.01	387.54	1.84	-0.632	0.631	3.158
11	2050	405	2.05	2038.24	393.12	2.01	0.574	2.933	1.951
12	2100	410	2.1	2085.33	394.07	2.07	0.699	3.885	1.429
13	2200	420	2.2	2169.76	395.26	2.22	1.375	5.890	-0.909
14	2300	430	2.3	2151.58	432.35	2.24	6.453	-0.547	2.609
15	2400	440	2.4	2373.22	444.81	2.48	1.116	-1.093	-3.333
16	2500	450	2.5	2351.07	447.13	2.49	5.957	0.638	0.400
17	2600	460	2.6	2428.93	448.39	2.62	6.580	2.524	-0.769
18	2700	470	2.7	2714.75	449.22	2.66	-0.546	4.421	1.481
19	2800	480	2.8	2728.42	473.86	2.83	2.556	1.279	-1.071
20	2900	490	2.9	2916.28	500.13	2.85	1.510	-2.067	1.724
