# Supplementary material for: Forward and reverse mapping for milling process using artificial neural networks
Source: Data Brief. 2017 Nov 4;16:114–21. doi: 10.1016/j.dib.2017.10.069 (PMC5694959; doi:10.1016/j.dib.2017.10.069)
Supplement: Supplementary file 1 — Supplementary material [file mmc1.docx]

**Conflicts of interest**

'Conflicts of interest: none'.
