# Supplementary material for: Forward and reverse mapping for milling process using artificial neural networks
Source: Data Brief. 2017 Nov 4;16:114–21. doi: 10.1016/j.dib.2017.10.069 (PMC5694959; doi:10.1016/j.dib.2017.10.069)
Supplement: Supplementary file 2 — Supplementary material [file mmc2.docx]

**Appendix A. Supplementary Material**

**Supplementary data associated with this article can be found in the online version at** [**https://link.springer.com/article/10.1007/s40430-016-0675-7**](https://link.springer.com/article/10.1007/s40430-016-0675-7)
